# Association between friendship quality and subjective wellbeing among adolescents: a systematic review

**DOI:** 10.1186/s12889-022-14776-4

**Published:** 2022-12-23

**Authors:** Abdullah Alsarrani, Ruth F. Hunter, Laura Dunne, Leandro Garcia

**Affiliations:** 1 Center for Public Health, Institute of Clinical Sciences, Royal Victoria Hospital, School of Medicine, Dentistry, and Biomedical Sciences, Queen’s University Belfast, Belfast, BT12 6BA Northern Ireland, UK; 2 College of Medicine, Taibah University, Medina, Saudi Arabia; 3 School of Social Sciences, Education, and Social Work, Queen’s University Belfast, Belfast, Northern Ireland, UK

**Keywords:** Friendship quality, Wellbeing, Life satisfaction, Mood, Happiness, Self-esteem

## Abstract

**Background:**

Social integration with friends has an important role in shaping adolescents’ behavior and determining their wellbeing. Friendship features such as companionship, trust, closeness, intimacy, and conflicts all form the concept of friendship quality. The quality of friendships can either enhance or impede mental development during adolescence. Therefore, this systematic review was conducted to understand the association between friendship quality and adolescents’ mental wellbeing.

**Methods:**

In November 2020 and later in August 2022, the search for evidence was conducted on five databases (Medline, Embase, ProQuest, Scopus, and PsycINFO). Only peer-reviewed quantitative studies published from January 2000 to August 2022 that investigated friendship quality as their exposure variable in relation to six constructs of subjective wellbeing (mood, loneliness, life satisfaction, happiness, self-esteem, and subjective wellbeing) were included. After screening for eligibility, two reviewers independently extracted the data based on population characteristics, study design, exposure and outcome variables, outcome measures used, and results. Risk of bias assessment was performed utilizing the NIH Quality Assessment Tool. Narrative evidence synthesis was performed based on the constructs of subjective wellbeing.

**Results:**

Forty-three articles out of 21,585 records were included in the review. The relationship between friendship quality and depression has been investigated extensively in the literature and negative (beneficial) associations were found in eighteen studies out of twenty-three. Poor peer relationship was associated with loneliness in nine studies out of ten. All seven studies on life satisfaction and quality of peer connection found a positive association. In five studies, better peer relationship was found to be associated with happiness. A positive association between friendship quality and self-esteem was observed in five out of six applicable studies. Friendship quality was found to be positively associated with subjective well-being in all of five included studies.

**Conclusions:**

Although majority of the included studies were cross-sectional in nature, this review demonstrates the paramount value of promoting healthy friendship to adolescents’ subjective wellbeing constructs. Interventions that aim to promote subjective wellbeing among adolescents should consider the development and maintenance of healthy friendships.

**Systematic review registration:**

PROSPERO CRD42020219312.

**Supplementary Information:**

The online version contains supplementary material available at 10.1186/s12889-022-14776-4.

## Introduction

Adolescents represent a considerable proportion of the human population, amounting to over one billion worldwide [1]. A crucial period of growth in all aspects of individual development, including psychological and social domains, occurs during adolescence age, which ranges from 10 to 19 years [1]. It is also a sensitive period as it determines the individuals’ intellectual abilities, social skills, and future behaviors, which need to be enhanced to ensure ideal transition to adulthood [1]. This stage of life carries its own risk as several health and behavioral issues develop during this important period, such as smoking initiation, illicit drug use, academic difficulties, unprotected sexual intercourse and its related outcomes, self-harm, and suicidal behavior [2]. Moreover, suicide and homicide are amongst the leading causes of mortality during adolescence [1]. Hence, adolescents’ wellbeing should be considered a priority by governments, public health agencies, and relevant stakeholders in order to mitigate anticipated challenges and enhance adolescents’ lives.

Several behavioral factors can either positively or negatively impact adolescents’ health and wellbeing. It has been known that poor diet [3], physical inactivity [4], inadequate sleep [5], tobacco use [6], and alcohol drinking [7] contribute to poor wellbeing outcomes. However, almost all behaviors can be influenced by social factors, particularly friendships. That is because friendship formation and socializing with same-age peers occupy a significant part in most adolescents’ social life [8]. Several studies have examined the role of friends in the adoption of unhealthy behavior and on developing negative wellbeing outcomes. For instance, Kim and Chun [9] found that friendship plays a key role on tobacco use among youth. It has also been evidenced that not only being involved with friends who are smokers affects one’s initiation of tobacco use, but also having no friends has an even greater impact on adoption of this behavior [10, 11]. Marijuana use between friends, if adopted, tended to be more mutually adopted and influenced by a user’s popularity [10, 12].

Furthermore, a study on youth population (with a mean age of 23.1 years, *n* = 183, 53.0% female), have found that alcohol consumption is increased with higher number of friends present in drinking occasions [13]. The same pattern of behavior and social effect was found on individuals’ meal choice and intake, which was similar to their friends’ food quality and quantity [14]. This phenomenon was more obvious between close friends. In the same regard, it was found that suicidal behavior between friends during adolescence occurs in the same manner, in which the likelihood of suicidal ideation and attempts is increased with exposure to friends’ suicidal behavior [15]. It was also found that adolescents who intentionally harmed themselves were more likely to had adopted this behavior from their friends [16]. Interestingly, this friendship effect on self-harm was observed among youths irrespective of their mental health status [16]. In another study, You et al. [17] addressed the moderating role of friendship characteristics and their impact on the relationship between psychological wellbeing and adoption of self-harm behavior. Additionally, work by Long et al. [18] has clarified how sharing of a certain risky behavior (i.e., disruptive behavior) among adolescents with mental health problems can play a role in the friendship formation and hence worsen their outcome.

The social interaction with friends is not limited to affecting adolescents’ behavior, it can also impact their subjective wellbeing. The area of how social support that adolescents received or perceived from their friends and significant others affect their wellbeing has been extensively researched. For instance, a meta-analysis study conducted by Chu et al. [19] have examined whether different social support sources and measures have an Impact on wellbeing of children and teens. They found that teacher and school personnel support have a greater value to wellbeing of children and adolescents than other sources of support, including friends support. Among the five measures of social support that the study categorized (size of social network, perceived social support, number of already enacted or received support, number of previously sought social support from others, other or undifferentiated measure of social support), perceived social support was the strongest measure linked to overall wellbeing (r = .201) while social network size was the weakest predictor (r = .01).

For instance, a longitudinal study conducted by Son and Padilla-Walker [20] have examined how different aspects of prosocial behavior towards friend among children and teens influence their relationships and impacts them psychologically. They found that in terms of quality, the more emotional support, in particular for girls, an adolescent perceives from their friends, the healthier their friendship outcomes and the lower their psychological distress in the future. In addition, Zhang et al. [21] suggest that youths should maintain healthy relationships and avoid conflicts with their peers and parents in order to have healthy mood. Moreover, Van Harmelen et al. [22] have demonstrated the protective role of social support from peers and parents against psychological distress for those who experienced early life stress. They found that higher perception of family and friend support at age 14 indirectly contribute to lower depressive symptoms at age 17 for those who have history of peer bullying and/or negative family environment.

Considering the important role of friends in shaping adolescents’ behavior and influencing their self-perception of wellbeing, ensuring they develop high-quality relationships is important. Berndt [23] claimed that friendship quality should not be confused with other characteristics of the friendship such as conflict, intimacy, companionship. He suggested that friendship quality should be dealt with as a global measure to describe the friendship either as rich or poor in quality regarding to how close to perfect the friendship features are. Another global measure that is important in adolescent life is subjective wellbeing. One’s subjective appraisal of his or her life in general as well as certain aspects of their life and activities is referred to subjective wellbeing [24]. According to Diener et al. [25, 26], subjective wellbeing is an important concept in assessing the health of community members. It encompasses an individual’s perception of happiness, feelings, mood, satisfaction in life and other important aspects of life (e.g., joy, affection, stress, and financial and future satisfaction). Accordingly, it is evident that several constructs make up and also influence the overall perception of subjective wellbeing. These are mood, life satisfaction, self-esteem, loneliness, and happiness. Each of these constructs has its own definition and unique value. The American Psychological association offered two psychological definitions of mood [27], each of them complements the other. Considering both of these definitions, mood can be defined as a self-perceived state of mind that varies in nature and degree which one may experience without an apparent reason but usually does not last for long time [28]. One may confuse mood with emotions, which are short-lived feelings, whereas mood is developed gradually and lasts longer [29, 30]. Depressed mood is linked to a number of negative outcomes during adolescence and young adulthood, such as poor academic performance [31], tobacco use [32], and early onset of alcohol use and its consequences [33]. Life satisfaction, the second construct, is an overall individual’s perception of their life quality that measures how satisfied someone is with his life [34]. Fostering life satisfaction among youth is crucial to promote their mental health, academic success, and healthy behavior [35, 36]. Regarding to happiness, which is referred to the perception of joy in life [37], one may experience happiness when their exposure to negative emotions is minimal and their perception of positive emotions, regardless of their intensity, occurs more frequently [38]. “Happiness is associated with and precedes numerous successful outcomes, as well as behaviors paralleling success” [39]. Loneliness construct can be described as one’s perception of being lonely or self-isolated as a result of not feeling integrated with people associated with [25, 26]. Loneliness can manifest at any age, particularly throughout adolescence, and is associated with poor mental health outcomes [40, 41]. One’s overall judgment and viewing on himself as a worthy human being is referred to self-esteem [34]. Higher levels of self-esteem not only help people achieve particular goals and alleviate the consequences of potential failure, it is also associated with better decision-making prior to failure [42]. It was found that people who reported having low levels of self-esteem during childhood and adolescence were more prone, albeit only to a small degree, to develop anxious and depressive symptoms in early adulthood [43].

It is known that reviews have been conducted that investigate certain aspects of friendship or types of friendship and their association with subjective well-being or other outcomes [19, 30, 44], but no manuscript was found that aimed to verify the association between friendship quality and subjective well-being outcomes in adolescents. A metanalysis conducted by Chu et al. [19] has examined various forms of social support and its impact on a broad range of children and adolescents’ wellbeing outcomes, including their mental state. They found that all areas of social support, including friendship support, that adolescents received or perceived are important to their mental health and wellbeing. Another systematic review conducted by Schacter et al. [44] looked at how friendship could play a positive role through buffering the relationship between exposure to bullying and mental health outcomes. The review failed to reach a conclusion due to a lack of consistency between the study results; the buffering effect was absent in some studies, while in the rest the results contradicted each other either in favor or against the existence of a moderating effect. On the other hand, a systematic review done by Webster et al. [30] investigated the role of social networking with friends either online or virtually on several subjective wellbeing outcomes. The study highlighted the beneficial effect of socializing with others rather than being isolated on adolescents’ self-esteem, loneliness perception, and mood, but not on body image. The study also successfully explained how subjective wellbeing outcomes are affected in the context of online social networking through excessive use of social media sites, exposure to direct negative comments, lack of interaction, and fear of not being up to date regarding friends’ posts or state.

Given all the above, it is clear that friendship support matters to adolescents’ wellbeing; however, the role that friendship quality can play on influencing subjective wellbeing outcomes among adolescents is still not well understood. The combined positivity and negativity effects model [45] suggests that the quality of all types of social relationships and subjective wellbeing are interrelated. According to this model, positive social relationships can influence wellbeing outcomes just as strongly as negative social relationships. Several studies supported this mode [46–48]. However, more empirical evidence is required to support this assumption, particularly for friendship quality among adolescents. Therefore, this systematic review aims to synthesize the evidence regarding the association between friendship quality and six constructs of subjective wellbeing in adolescents.

## Methods

Prior to the initial search, the review was registered at PROSPERO (registration number CRD42020219312). We followed PRISMA [49] and SWiM reporting guidelines [50] in this work (for more details, see Additional files 1, 2 and 3). On 10 November 2020 (and later on 18 August 2022), a systematic search of five databases (Medline, Embase, ProQuest, Scopus, and PsycINFO) was conducted using the following keywords and their synonyms: friend, peer relation, loneliness, life satisfaction, mood, happiness, wellbeing, self-esteem, quality of life, adolescent, teen, and youth (see Table 1). The search was constructed after consultation with a subject librarian at the Center for Public Health at Queen’s University Belfast. Due to the expansion of survey and research methods in this area over the last two decades, and to be up to date with the current knowledge and practice relevant to the current generation, the search was restricted to articles published from January 2000 to August 2022. Only peer-reviewed English articles were included in the review. Materials that fell in one of the following categories were excluded from the review: qualitative articles, review articles, dissertations, books, reports, conference, and editorial papers. Only studies that measured global friendship quality as their exposure variable were included. Papers that investigated individual facets of friendship quality (e.g., closeness, companionship, trust, and conflict) were excluded. The outcomes could be any of the following subjective wellbeing outcomes: self-esteem, happiness, mood, life satisfaction, loneliness, and subjective wellbeing. Articles must have quantitatively investigated the relationship between friendship quality and at least one of the preidentified wellbeing outcomes. An article was included if it had a mean population age between 10 to 19 years. No restrictions were set on the health status of the population.

**Table 1:** Search terms used in databases

	Population	Exposure	Outcome
Used Keywords	Adolescen* OR teen* OR youth*	Friend* OR peer relation*	Wellbeing OR well-being OR mood OR lon* OR self-esteem OR “life satisfaction” OR “quality of life” OR happ*

The above-mentioned search strategy and selection criteria are different from what was originally set in the PROSPERO record (CRD42020219312) in two ways. First, we considered to include studies that investigated individual features of friendship quality (e.g., closeness, intimacy, and conflicts), but with further discussion we decided to amend the study protocol and only include studies that used a global measure of friendship quality. This decision was based on the definition of friendship quality offered by Berndt [23], which suggests that friendship quality should be differentiated from friendship features and viewed as a single construct to facilitate the judgment of the quality of any friendship. Second, we originally considered to include qualitative as well as quantitative original studies, but then decided to exclude qualitative ones to make data synthesis more consistent by focusing only on one class of research methods. Both changes in the protocol happened during the titles and abstracts screening phase.

Articles’ titles and abstract were screened against the eligibility criteria by one reviewer. The second reviewer screened 10% of the articles (1455 records) to establish the quality of screening at this stage and ascertain the level of agreement. Divergences happened in only 18 articles (1.2%), mostly in the direction of increased sensitivity (i.e., the first reviewer did not exclude an article that the second reviewer would have it). The conflicts were settled through discussion.

For full-text review, a data extraction form was developed by the first author and two colleagues, and the following items were extracted for each of the included studies: number of the participants, mean age, date of the study, type of the study, outcome(s), type of exposure, country in which the study took place, and the study finding(s). The data extraction was completed by the first author and the quality of the extraction was ascertained by double-checking the entire extraction process by a second reviewer, with disagreements settled through discussion and consultation with a third independent researcher. Along with the interpretation of the study finding in the data extraction form, the direction of the significant statistical association found in each study was denoted by “+ve” or “-ve” (positive and negative, respectively), while “null” was added if no significant association was found. In descriptive studies, where the comparison between groups is based on the difference in means or proportions in the outcome, only a brief description of the result was given.

The methodological quality of each study was assessed using The National Institutes of Health (NIH) Quality Assessment Tool for Observational Cohort, Cross-Sectional, and Case-Control Studies [51]. Although this tool is not standardized, it gives the researchers the freedom to set their own parameters and hence better judgment on quality of a study can be achieved [51]. Several systematic review studies employed this tool and benefited from its flexibility [52–54]. The tool contains 14 questions to assess the quality of cohort and cross-sectional studies, while the case-control studies’ tool has 12 questions. These questions were designed to capture potential methodological flaws in the included studies. The main focus of these questions is on whether a paper properly identified, explained, measured, and/or provided information about the following: research objectives, study population, sample size justification, participation rate, exposure and outcome variables and timeframe between them, adjusted for confounding variables, and dropout. For the purpose of this review, three items were added to the quality assessment tool, with one point in the final score added for an item if the answer was “yes”. These items are: the study is not limited to a very specific population group (i.e., lack of generalizability to general adolescent population); use of validated measure for each exposure and outcome variable, and the study is not descriptive or correlational in nature. The quality assessment was done by one researcher. After adding those elements to the assessment tool, cross sectional and cohort studies were assessed on a scale of 0 to 17, while case-control studies were assessed on 0 to 15 scale (for more details, see Additional file 4).

The evidence was synthesized based on the six preidentified constructs of subjective wellbeing under investigation. The narrative description of the results focused on the consistency of the findings between studies, while taking into account the study design, methodological quality, and the generalizability of the findings according to the characteristics of the sample of the included studies.

## Results

**Fig. 1:**
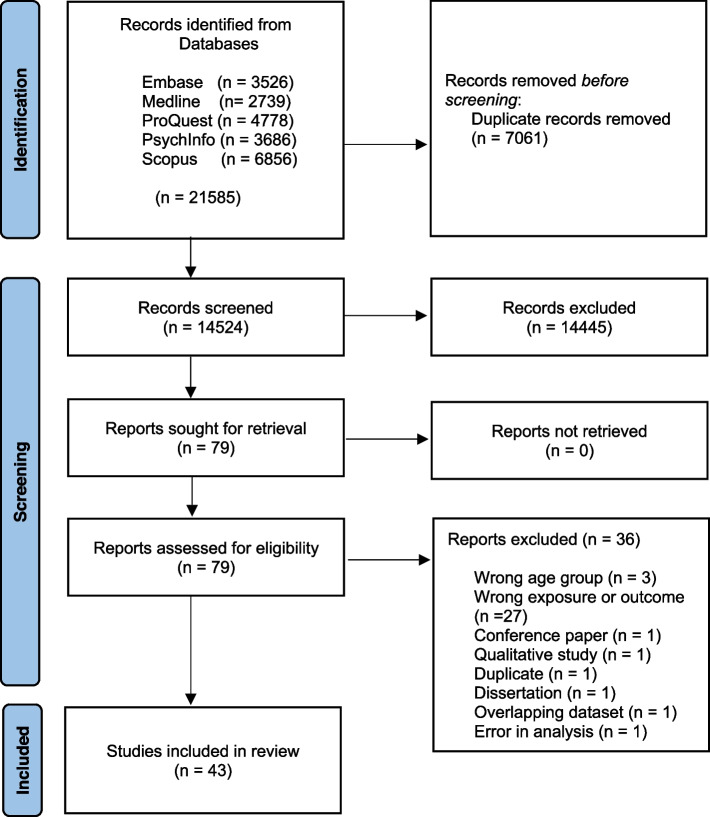
Data screening and extraction stages

A total of 21,585 articles were found in the five databases, out of which 14,524 articles remained after duplicates were removed. Title and abstract screening resulted in the exclusion of 14,481 irrelevant articles. Seventy-nine articles were deemed eligible for full text screening, of which 36 papers, the majority because the exposure variable, were not eligible for our systematic review (e.g., the use of only one domain of friendship quality scales or multidimensional measures that combine relationship quality for friends and significant others). Thus, 43 articles were included in the final analysis and data extraction [4, 55–98]. Figure 1 shows the steps of the data screening and extraction. Table 2 shows a breakdown of the included studies by outcome and study design along with a brief summary of the findings for each subjective wellbeing constructs.

**Table 2:** Summary of findings per subjective wellbeing constructs, methadological quality index

Wellbeing outcome	Number of studies per analysis type	Findings	Methadological quality index range
**Depression (23 studies)**	Cross-sectional: 16 studies [4, 57–67, 88, 93, 95, 97].	Fourteen studies found a significant benificial association between friendship quality and depressive symptoms, while two found no association.	6–15
	Longitudinal: 6 studies [83–86, 92, 94, 98].	Four studies found a significant beneficial relationship between friendship quality and depressive symptoms; two studies found a bidirectional relationship; and one study found no effect.	12–14
	Case- Control: 1 study [56].	The study found no significant association between friendship quality and depressive symptoms, neither in adolescents with Asperger’s syndrome nor in typically developed groups.	7
**Loneliness (10 studies)**	Cross-sectional: 8 studies [62, 67–72, 93].	Seven studies found a significant beneficial association between friendship quality and loneliness, while only one study found no effect.	8–15
	Case-control: 2 studies [55, 56].	One study found a significant beneficial association between friendship quality and loneliness among typically-developed adolescents but not among adolescents with Asperger’s syndrome, while the other study showed that lonileness score was higher among adolescents with ASD who had poor peer relationships.	7–8
**Life satisfaction (8 studies)**	Cross-sectional: 7 studies [73–77, 89, 91].	All seven studies found a significant positive association between the quality of friendships and life satisfaction.	8–10
	Longitudinal: 1 study [90].	The study found a significant positive longitudinal association between friendship quality and life satisfaction.	13
**Self-esteem (6 studies)**	Cross-sectional: 6 studies [66, 67, 78–80, 87, 93].	All but one study found a significant positive association between the friendship quality and self-esteem.	7–10
**Happiness (5 studies)**	Cross-sectional: 4 studies [63; 74; 69, 60].	All four studies found a significant positive association between friendship quality and happiness.	8–11
	Longitdinal: 1 study [83, 86].	The study found a significant positive longitudinal association between friendship quality and happiness.	13
**Subjective wellbeing (5 studies)**	Cross-sectional: 4 studies [78, 79, 82, 91]	All four studies found a significant positive association between friendship quality and subjective wellbeing.	9
	Longitudinal: 1 study [96].	The study found no significant longitudinal association between friendship quality and happiness.	12

Of the 43 articles included (Table 3), 31 were cross-sectional [4, 57–60, 62–82, 87–89, 91, 95, 97] and only 10 longitudinal [61, 83–86, 90, 92–94, 96, 98] and 2 case-control studies were found [55, 56]. Depressive symptoms were the most investigated wellbeing outcome (23 studies: [4, 56–67, 83–86, 88, 92–95, 97, 98]), followed by loneliness (10 studies: [55, 56, 62, 67–72, 93]), life satisfaction (8 studies: [73–77, 89–91]), self-esteem (6 studies: [66, 67, 78–80, 87, 93]), happiness (5 studies: [67, 70, 76, 81, 83, 86]), and subjective wellbeing (5 studies: [78, 79, 82, 91, 96]).

**Table 3:** Study characteristics, findings, and methodological quality

Abbreviated reference	Country	Study design	Setting / sample characteristics	Exposure	Outcome	Findings	Study quality score
Field et al., [4]	United States	Cross-sectional	Setting: school *N* = 79 (% female not reported) Age (range): high-school seniors (17–18 years)	1*Relationship with parents/friends* were assessed with questions adapted from previous study [99]	*Depression* was assessed using the Center for Epidemiological Studies Depression Scale (CES-D [100]).	Depressed adolescents (CES-D ≥ 19) reported inferior levels of friendship quality, fewer friends, and being less popular.	6
De Matos et al., [57]	Portugal	Cross-sectional	Setting: school *N* = 6903 (53% female) Age (mean): 14.1 years (SD: 1.71)	*Peer relationships* were assessed with questions adapted from the Portuguese version of Health behavior in School-Aged Children (HSBC) 1998 survey.	1*Depression* was assessed with 2 questions.	Participants classified as depressed had significantly lower mean scores for peer relationships.	7
Kullik and Petermann [58]	Germany	Cross-sectional	Setting: school *N* = 248 (51.2% female) Age (mean): 14.4 years (SD: 1.39)	*Peer attachment* was assessed using the German short version of Inventory of Parent and Peer Attachment [101].	*Depression* was measured using the German version of the CES-D [102].	-ve: Depressive symptoms were significantly correlated with attachment to peers for both females (β = −.31, *p* < 0.001) and males (β = −.21, *p* < .05). Internal- and external-functional emotion regulation were used as mediators. For females, internal-functional emotion regulation partially mediated the relationship between attachment to peers and depressive symptoms. For males, both internal- and external-functional emotion regulation partly mediated the relationship between peer attachment and depressive symptoms.	9
Fadda et al., [73]	Italy	Cross-sectional	Setting: school *N* = 1193 (50.3% female) Age (mean): 16.85 years (SD: 1.74)	*Relationship with peers* was assessed with three items from the Health Behaviour Questionnaire [103]	*Positive psychological functioning* was assessed using the revised Oxford Happiness Inventory (OHI [104])	+ve A direct significant positive association was found between peer relationships and life satisfaction (β = .17, *p* < .01). An indirect positive association was also observed for peer relationships and life satisfaction partially mediated by self-esteem (β = .06, *p* < .01).	9
Wong [59]	United states	Cross-sectional	Setting: school *N* = 144 (50.7% female) Age (mean): 15.7 years (SD: 1.35)	*Peer attachment* was assessed using the Inventory of Parent and Peer Attachment (IPPA [101]).	*Psychological wellbeing* was assessed with the CES-D.	-ve Peer relationships were negatively correlated with depressive symptoms (β = −.29, *p* = .01). Participants who reported more positive peer relationships experienced lower levels of depression.	8
Afifi et al., [60]	Oman	Cross-sectional	Setting: school *N* = 5409 (49.7% female) Age (mean): 17.11 years	*Relationship with friends*	*Depressive symptoms* were measured using the Child Depression Inventory [105].	-ve Positive friendship quality was significantly associated with lower odds of having at least mild depressive symptoms (OR = 0.69, 95% CI: 0.63–0.76).	8
Vanhalst et al., [68]	Belgium	Cross-sectional	Setting: school *N* = 884 (68% female) Age (mean): 15.79 years (SD: 1.33)	*Friendship quality* was assessed with the Friendship Qualities Scale (FQS [106]). the other one of us, we *Self-esteem* was measured using the Rosenberg Self-esteem Scale [107]. Shyness was measured using peer nomination with participants selecting other members of their class who they regarded as “shy and/or socially withdrawn”. Received nominations were counted for each individual. Friendship quality was used as a direct predictor and as a mediator between loneliness and self-esteem and shyness.	*Peer-related Loneliness* was assessed using the Loneliness and Aloneness Scale for Children and Adolescents (LACA [108]).	-ve: Friendship quality was significantly negatively associated with loneliness (β = −.24, *p* < .001). Friendship quality acted as a partial mediator between shyness and loneliness (β = .03, 95% CI: 0.01–0.05), and also between self-esteem and loneliness (β = −.07, 95% CI: − 0.11--0.04).	10
Whitehouse et al., [56]	Australia	Case-control	Setting: school **Cases**: 35 adolescents with Asperger’s Syndrome (AS), (17.1% female) Age (mean): 14.2 (SD: 0.80) Controls: 35 adolescents (20% female) Age (mean): 14.4 (SD: 0.10)	*Friendship quality* was assessed using the Friendship Quality Questionnaire (FQQ [109]).	*Loneliness* was measured using the De Jong-Gierveld Loneliness Scale [110]. *Depressive symptoms* were measured using the children’s version of the CES-D (CES-DC) [111].	*Friendship quality and loneliness*: -ve In the Typical group, overall friendship score was negatively associated with loneliness (r = − 0.36, *p* < 0.05). This was the same for the AS group, but the strength of the association was smaller (r = − 0.28, *p* < 0.05). In the regression analysis, overall friendship score predicted levels of loneliness in the Typical group (R^2^ = 0.12, F = 4.84, *p* < 0.05) but not the AS group. *Friendship quality and depressive symptoms*: Overall friendship quality did not predict depressive symptoms in either group.	7
Jose, [83, 86]	New Zealand	Longitudinal	Setting: school *N* = 1774 (52% female) Age (mean): 12.21 (SD: 1.75) Period: one year	*Peer connectedness* was assessed with seven items [112].	*Positive affect* or *happiness* was assessed with the CES-D. *Negative affect* or *depressive symptoms* was assessed the CES-D.	*Peer connectedness and positive affect (happiness)*: +ve Peer connectedness at T1 was significantly positively associated with positive affect at T2 (β = 0.12, *p* = 0.0002). This relationship was bidirectional, with individuals who reported higher positive affect at T1 also reported an increase in peer connectedness at T2 (β = 0.05, *p* = 0.037). *Peer connectedness and negative affect (depressive symptoms)*: -ve Peer connectedness was significantly negatively associated with negative affect (β = − 0.07, *p* = 0.011).	13
Oberle et al., [74]	Canada	Cross-sectional	Setting: school *N* = 1402 (47% female) Age (mean): 14.6 (SD: 1.03)	*Positive peer relationships* was assessed using the Resilience Inventory [113, 114].	*Life satisfaction* was assessed using the Satisfaction with Life Scale for Children (SWLS-C [115]).	+ve Positive peer relationships were positively associated with life satisfaction (β = 0.12, t = 4.00, *p* < 0.001).	10
Lodder et al., [69]	Netherlands	Cross-sectional	Setting: school *N* = 1172 (50.9% female) Age (mean): 12.81 (SD: 0.43)	*Best friendship quality* was assessed using the Investment Model Scale [116]. friendship quality (α = 0.88). *Network friendship quality* was assessed using the Peer subscale of the IPPA.	*Loneliness* was assessed using the Louvain Loneliness and Aloneness Scale for Children and Adolescents [108].	-ve *Best Friendship Quality and Loneliness*: Best friendship quality was negatively associated with loneliness (β = −.24, *p* < 0.001). *Network friendship quality and loneliness*: Network friendship quality was negatively associated with loneliness (β = −.45, *p* < 0.001).	10
Lambert et al., [81]	New Zealand	Cross-sectional	Setting: school *N* = 9107 (46.4% female) Age (range): 13 or under – 17 and over (SD: n/a)	*Friend/peer connection* was assessed with nine items.	*Happiness* was measured using the WHO-5 Wellbeing Index [117].	+ve: Friend/peer connection was positively associated with happiness (β = 1.49, *p* < 0.0001).	11
Laghi et al., [75]	Italy	Cross-sectional	Setting: school *N* = 1211 (59.6% female) Age (mean): 17.31 (SD: 1.09)	*Peer attachment* was assessed with the IPPA.	*Life satisfaction* was assessed using the Satisfaction with Life Scale (SWLS [118]).	+ve Peer attachment was moderately positively associated with life satisfaction (β = 0.09, *p* < 0.01).	9
Sasikala and Cecil, [78]	India	Cross-sectional	Setting: school *N* = 97 (59.8% female) Age (mean): 16.9 (SD: n/a)	*Peer attachment* was measured using the IPPA.	*Self-esteem* was measured using the Rosenberg Self-esteem Scale. *Psychological wellbeing* was measured using the General Health Questionnaire [119].	+ve Peer attachment was positively correlated with self-esteem (β = 0.23, *p* < 0.05) and subjective wellbeing (β = 0.37, *p* < 0.01).	9
Balluerka et al., [79]	Spain	Cross-sectional	Setting: school *N* = 2182 (51.6% female) Age (mean): 14.51 (SD: 1.55)	*Peer attachment* was assessed using the IPPA.	*Positive affect of psychological wellbeing* was assessed using the Children’s Depression Scale (CDS [120]).	+ve Peer attachment was significantly positively associated with positive affect (β = 0.16, *p* < 0.0001).	9
Biggs et al., [61]	United States	Longitudinal (but the relationship under investigation was assessed cross-sectionally)	Setting: school N = 91 (56.5% female) Age (mean): 15.5 (SD: 0.61)	*Friendship quality* was assessed using a short version of the FQQ.	*Depression symptoms* were assessed using the short form of the Children’s Depression Inventory (CDI-SF [121]).	Null Positive friendship quality and friendship conflict were not significantly associated with depression at T2.	11
Al-Yagon, [70]	Israel	Cross-sectional	Setting: school *N* = 280 (55% female) Age (mean): 15.94 (SD: 0.70) Participants were categorized into three groups: (i) adolescents with learning disability (LD); (ii) adolescents with LD and attention-deficit hyperactivity disorder (LD-ADHD); and (iii) typically developed adolescents (TD).	*Friendship quality* was assessed using the Hebrew adaptation of the FQQ [122].	*Loneliness* was measured using the Peer-Network Loneliness and Peer-Dyadic Loneliness Scale [123]. *Positive affect/happiness* was assessed using the Hebrew adaptation of the Affect Scale ( [61, 124, 125].	-ve Adolescents (TD) who reported higher friendship quality reported significantly lower peer-network/peer-dyadic loneliness (β = − 0.40 and β = − 0.53 respectively). In the other groups (LD, LD-ADHD), friendship quality was significantly negatively associated with peer-network/peer-dyadic loneliness (LD: β = − 0.37 and β = − 0.48 respectively; LD-ADHD: β = − 0.42 and β = − 0.59 respectively). A significant association was found between positive affect and friendship quality in the LD-ADHD group only (β = 0.65).	10
Corsano et al., [71]	Italy	Cross-sectional	Setting: school *N* = 330 (50.9% female) Age (mean): 15.04 (SD: 2.47)	*Interpersonal relationships* were assessed using the Italian adaptation of the Assessment of Interpersonal Relations [126].	*Loneliness* was assessed using the Italian adaptation of the Louvain Loneliness Scale for Children and Adolescents [127].	-ve: Interpersonal relations with male peers was significantly negatively associated with peer loneliness (r = − 0.16, *p* < 0.01). Interpersonal relations with female peers was significantly negatively associated with peer loneliness (r = − 0.19, *p* < 0.001).	8
Kamper and Ostrov, [84]	United States	Longitudinal	Setting: school *N* = 776 (49.6% female) Age (mean): 11 (SD: 0.23) Duration: 4 years (from age 11 to 15 years)	*Friendship quality* was assessed with the FQQ. Positive and negative friendship quality were investigated as potential mediators in the relationship between relational aggression and depressive symptoms.	*Depressive symptoms* were assessed using the CDI-SF.	+ve Negative friendship quality positively predicted an increase in depressive symptoms (β = 0.31, *p* < 0.05). Negative friendship quality also acted as a partial mediator between relational aggression and depression (F = 16.40, *p* < 0.001, R^2^ = 0.10).	13
Lieb and Bohnert, [62]	United States	Cross-sectional	Setting: online groups and healthcare settings *N* = 127 adolescents with autism spectrum disorder (ASD) (18.9% female) Age (mean): 13.95 (SD: 1.60)	*Friendship quality* was assessed using the abbreviated version of the FQQ (FQQ-A). Friendship quality was also examined as a potential mediator between executive functions and loneliness and depressive symptoms.	*Loneliness* was assessed with the Children’s Loneliness and Social Dissatisfaction Scale [128]. *Depressive symptoms* were assessed with Achenbach Youth Self Report Depression Scale (YSR-D; [129]).	*Friendship quality and loneliness*: -ve The results indicated that there was a significant negative bivariate correlation between friendship quality and loneliness (r = − 0.43, *p* < 0.01). Friendship quality was also negatively associated with loneliness in a separate mediation model (β = − 0.26, *p* ≤ 0.001). Friendship quality mediated the relationship between emotional control and loneliness (β = 0.65, p ≤ 0.001; Sobel test = 0.12, *p* ≤ 0.05). *Friendship quality and depressive symptoms*: null No significant relationship was found between friendship quality and depressive symptoms.	15
Raboteg-Saric and Sakic, [76]	Croatia	Cross-sectional	Setting: school *N* = 401 (39.9% female) Age (mean): 16.92 (SD: 1.16)	*Friendship quality* was assessed with the short form version of the FQS [130].	*Happiness* was measured using the Subjective Happiness Scale (SHS [131]). *Life satisfaction* was assessed using the Student’s Life Satisfaction Scale (SLSS [132]). *Self-esteem* was assessed using the Rosenberg Self-Esteem Scale.	*Friendship quality and happiness*: Adolescents who reported higher levels of friendship quality also had greater levels of happiness (F = 18.43, *p* < 0.001) partial η^2^ = 0.06). *Friendship quality and life satisfaction*: Adolescents with higher levels of friendship quality were more satisfied with life (F = 4.22, *p* = 0.041, partial η^2^ = 0.01). *Friendship quality and self-esteem*: Adolescents who rated their friendship quality more favourably had marginally higher levels of self-esteem (F = 3.42, *p* < 0.065, η^2^ = 0.01).	8
Oppenheimer and Hankin, [85]	United States	Longitudinal Data collection at three time points across five-week intervals	Setting: school *N* = 350 (57% female) Age (mean): 14.5 (SD: 1.40)	*Relationship quality* was assessed using the short version of the Network of Relationships Inventory (NRI; [133]).	*Depressive symptoms* were assessed using the CDI.	Null: Adolescents’ perception of friendship quality has no effect on their depressive symptoms while the inverse is true Standardized path coefficients indicated that depressive symptoms (T1) were significantly negatively associated with positive relationship quality (T2) (β = − 0.13, p ≤ 0.001) and positively associated with negative relationship qualities (T2) (β = 0.14, *p* ≤ 0.01). Likewise, depressive symptoms (T2) were significantly negatively associated with positive relationship quality (T3) (β = − 0.15, p ≤ 0.001) and positively associated with negative relationship qualities (T3) (β = 0.07, p ≤ 0.01).	13
Huang and Chen, [63]	Taiwan	Cross-sectional	Setting: school *N* = 1325 (53.2% female) Age (mean): 16.5 (SD: 0.90)	*Negative peer relationships* were assessed using the Taiwanese Relationship Inventory for Child and Adolescent [134].	*Depressed mood* was assessed using Chinese version of the Center of Epidemiological Studies Scale [135].	+ve: The results indicated a significant positive bivariate correlation between negative peer relationships and depressed mood (r = 0.49, *p* < 0.001). Result of the multiple linear regression model showed a significant positive association between negative peer relationships and depressed mood (β = 0.28, *p* < 0.001).	11
MacPhee and Andrews, [64]	Canada	Cross-sectional	Setting: home *N* = 2014 Female percentage (n/a) Age (range): 12–13	*Peer relations* were assessed using the Peer Relations scale of the Self-Description Questionnaire (SDS [136]).	*Depressive symptoms* were assessed using a shortened version of the CES-D.	+ve Peer relations were significantly associated with depressive symptoms in the multivariate stepwise regression analysis (R^2^ = 0.418, ∆ R^2^ = 0.002, *p* < 0.001). For males, peer relations were significantly associated with depressive symptoms (R^2^ = 0.352, ∆ R^2^ = 0.013, *p* < 0.001), however, this was not the case for females.	10
Newland et al., [77]	United States	Cross-sectional	Setting: school *N* = 149 (47.7% female) Age (mean): 13 (SD: n/a)	*Peer relationships* were assessed with an adapted version of the Children’s Worlds survey [137]	*Life satisfaction* was assessed with the Children’s Worlds survey (α = 0.90).	+ve: The results showed a significant positive bivariate correlation between peer relationships and life satisfaction (r = 0.59, *p* < 0.001). Results from the regression analysis indicated that adolescents who reported higher quality peer relationships had higher levels of life satisfaction (β = 0.32, *p* < 0.001).	10
Preddy and Fite, [65]	United States	Cross-sectional	Setting: home *N* = 89 (44.0% female) Age (mean): 10.4 (SD: 1.1)	*Friendship quality* was assessed with an abbreviated version of the FQQ to assess friendship quality with a best friend. Friendship quality was also examined as a moderator between different subtypes of aggression and depressive symptoms.	*Depressive symptoms* were assessed using the CDI.	-ve: The results indicated that there was a significant negative bivariate correlation between friendship quality and depressive symptoms (r = − 0.25, *p* < 0.05). Regression analysis results indicated that friendship quality was negatively associated with depressive symptoms (β = − 0.11, *p* = 0.02). Friendship quality did not moderate the relationship between aggression subtypes and depressive symptoms.	11
Ang, [72]	Malaysia	Cross-sectional	Setting: school *N* = 618 (57.0% female) Age (mean): 14.80 (SD: 0.99)	*Friend connectedness* was measured using the Hemingway Measure of Adolescent Connectedness (MAC [138]).	*Loneliness* was assessed using the UCLA Loneliness Scale [139]	Null The results indicated no statistically significant association between friend connectedness and loneliness (β = − 0.02, *p* > 0.05).	11
Chang et al., [55]	Taiwan	Case-control	Setting: school, home, clinic, and community Cases: 101 adolescents with ASD (20.2% female) Age (mean): 15.6 (SD: n/a) Controls: 101 neuro-typical adolescents (74.5% female) Age (mean): 16.1 (SD: n/a)	*Friendship quality* was assessed using the FQS	*Loneliness* was assessed using the Chinese version of the short-form UCLA Loneliness Scale [134].	-ve: The results showed a significant negative bivariate correlation between friendship quality and loneliness for the ASD group (r = − 0.229, *p* < 0.05) and neuro-typical group (r = − 0.220, *p* < 0.05).	8
McMahon et al., [82]	Ireland	Cross sectional	Setting: Home *N* = 7527 (51.1% female) Age (specific): 13 (98.4% of the sample) (SD: n/a)	*Peer attachment* was assessed with the IPPA.	*Psychological wellbeing* was assessed with The Piers-Harris Self-Concept Scale [140]	-ve Friendship quality was significantly associated with subjective wellbeing for (β = − 1.00, *p* < .001, [− 1.57, −.44], R2 = .006) but not for boys (β = −.27, *p* = .37, [−.85, .31]). For girls, friendship quality partially meditated the association between stressful life events and subjective wellbeing (β = −.27, SE = −.27, [−.51, −.03]) For boys, friendship quality has no mediation effect on the association between stressful life events and psychological wellbeing	9
Smokowski et al., [66]	United States	Cross sectional	Setting: home *N* = 4321 (53.0% female) Age (mean): 12.8 (SD: n/a)	*Friendship quality* was assessed using the Negative peer relationships scale [141].	*Depressive symptoms* were assessed using the School Success Profile (SSP) [141]. *Self-esteem* was assessed using the Rosenberg Self-Esteem Scale.	*Negative peer relationships and Depressive symptoms:* Results indicated that adolescents who reported higher negative peer relationships had higher levels of depressive symptoms (*p* < 0.001). *Negative peer relationships and self-esteem*: Adolescents who reported higher negative peer relationships had lower levels of self-esteem (*p* < 0.001).	8
Nyarko, [80, 87]	Ghana	Cross sectional	Setting: school *N* = 100 (34.0% female) Age (range): 15–18 (SD: n/a)	*Peer relationship* was measured using the revised class play method of peer assessment [142].	*Self-esteem* was assessed using the Rosenberg Self-Esteem Scale..	-ve: The results showed a significant negative correlation between friendship quality and self-esteem (r = − 0.231, *p* < 0.05)	7
Spithoven et al., [67]	Belgium and Netherlands	Cross sectional	Two samples were used in this study but only one sample falls under our eligibility criteria Sample of Dutch adolescents – 2nd sample): Setting: school *N* = 1361 (51.32% female) Age (mean): 12.81 (SD: 0.42)	*Friendship quality* was assessed with theIPPA.	*Depressive symptoms* were measured using short form of the (CES-D [143]). *Peer-related Loneliness* was assessed using the LACA. *Self-esteem* was assessed using the Single Item Self-Esteem scale [144]. *Happiness* was assessed using a single item scale [145].	*Friendship quality and depressive symptoms:* -ve: Friendship quality inversely correlated with depressive symptoms (r = − 0.39, *p* < 0.001) *Friendship quality and loneliness:* -ve: Friendship quality significantly negatively associated with loneliness (r = − 0.52, *p* < 0.001) *Friendship quality and self-esteem:* +ve Friendship quality positively associated with self-esteem (r = 0.21, *p* < 0.001) *Friendship quality and happiness:* +ve Friendship quality positively correlated with happiness (r = 0.36, *p* < 0.001)	9
O’Connor et al., [88]	Netherlands	Cross-sectional	Setting: Autism center, special education school, and public school *N* = 306 Age (mean): 11.69 (SD: 1.33) Autistic group: *N* = 104 (17.3% female) Typically developed group: *N* = 202 (55.0% female)	*Friendship Quality* was assesses using the Best Friend Index (BFI [146];	*Depressive symptoms* were assessed using the Dutch version of CDI [147]	For females, negative friendship quality was positively associated with depressive symptoms in autistic (r = 0.48, 95% CI: 0.02–0.77) and typically developed adolescents (r = 0.28, 95% CI: 0.10–0.44), whereas positive friendship quality was negatively correlated among typically developed group only (r = − 0.24, 95% CI: − 0.41 - -0.06) For males, positive friendship quality was negatively correlated with depressive symptoms only among autistic group (r = − 0.37, 95% CI: − 0.54 - -0.17)	7
Putri and Muttaqin, [89]	Indonesia	Cross-sectional	Setting: online survey *N* = 450 (70.4% female) Age (mean): 16.0 (SD: 2.58)	*Friendship Quality* was assessed using McGill Friendship Questionnaire-Friend’s Functions (MFQ-FF [148])	*Life Satisfaction* was assessed using Satisfaction with Life Scale (SWLS: [149])	Positive associations between friendship quality and life satisfaction were observed via four indirect paths: 1) Basic psychological need satisfaction (β = .330, *p* < 0.001) 2) Autonomy (β = 0.183, *p* < 0.001) 3) Competence (β = 0.224) 4) Relatedness (β = 0.303, *p* < 0.001)	9
Lim, [90]	South Korea	Longitudinal	Setting: School, home *N* = 2250 (46.6% female) Age (mean): 14.0 (SD: 0.03)	*Negative peer relationships* were assessed using Peer Relationship Quality Scale [150]	*Life satisfaction* was assessed using SWLS.	Standardized path coefficients indicated that negative peer relationships (T1) were significantly negatively associated with life satisfaction (T2) (β = − 0.043, *p* < 0.01). Standardized path coefficient also indicated a significant negative relationship between negative peer relationships (T2) and life satisfaction (T3) (β = − 0.084, *p* < 0.01). Negative peer relationships quality (T2) acted as a full mediator between smartphone dependence (T1) and life satisfaction (T3) (β = − 0.043, 95% CI: − 0.082- -0.020)	13
Kühner et al., [91]	China	Cross-Sectional	Setting: School *N* = 1279 (47.8% female) Age (mean): 11.47 (SD: 1.12)	*Peer relationship* was assessed using four questions developed and tested by the author	*Subjective wellbeing was assessed using* Children’s Worlds Subjective Well-Being Scale (CW-SWBS [151]) *Overall life satisfaction* was assessed using CW-SWBS	*Peer relationship and subjective wellbeing:* Overall peer relationship score was positively associated with subjective wellbeing (β = .150, *p* < .001) Peer relationship quality acted as a partial mediator between socioeconomic status and subjective wellbeing (β = 0.011, 95% CI: 0.003–0.021) *Peer relationship and overall life satisfaction:* Overall peer relationship score was positively associated with subjective wellbeing (β = .090, *p* < .001) Peer relationship quality acted as a partial mediator between socioeconomic status and overall life satisfaction (β = 0.007, 95% CI: 0.001–0.014)	9
Choe and Yu, [92]	South Korea	Longitudinal	Setting: School *N* = 1737 (48.4% female) Age (mean): 14.0 (SD: 2.0)	*Friendships* was assessed using the School Life Adaptation Scale—Peer Relationships [152]	*Depressive symptoms* were assessed using Depression Scales of the Korean Mental Diagnostic Test [153]	Longitudinal bidirectional relationship between friendships and depressive symptoms was observed Significant inverse relationships were found between friendships (T1) and depressive symptoms (T2) (β = − 0.148, *p* = 0.000), and inverse was true (β = − 0.091, p = 0.000) Significant inverse relationships were found between friendships (T1) depressive symptoms (T2) (β = − 0.109, p = 0.000), and the inverse was true (β = − 0.066, p = 0.000).	13
Forgeron et al., [93]	Canada	Longitudinal (but the relationship under investigation was assessed cross-sectionally)	Setting: healthcare *N* = 83 (% female not reported) Age (mean): 15.29 (SD: 1.26)	*Friendship quality* was assessed using the FQS.	*Depressed mood* was assessed using the CES-D. *Loneliness* was assessed using the Loneliness Scale [128]. *Self-esteem* was assessed the Rosenberg Self-Esteem Scale.	*Friendship quality and depressed mood:* -ve The results indicated that there was a significant bivariate correlation between depressed mood (T2) and negative (r = − 0.311, *p* < 0.01) and positive (r = − 0.293, *p* < 0.01) friendship quality (T2), but no significant relationships were identified between the same variables at T1 *Friendship quality and loneliness:* -ve There was a significant negative bivariate cross-sectional correlation solely between negative friendship quality and loneliness for each wave (T1: r = − 0.223, p 0.05; T2: r = − 0.270, p 0.05; T3: r = − 0.223, p 0.05). *Friendship quality and self-esteem:* +ve The results indicated that there was a significant positive bivariate correlation between self-esteem (T2) and negative (r = 0.296, *p* < 0.01) and positive (r = 0.270, *p* < 0.05) friendship quality (T2), but no significant relationships were identified between the same variables at T1	10
Powell et al., [94]	United Kingdome	Longitudinal	Setting: School *N* = 1712 (46.5% female) Age (range): 11–12	*Friendship quality of the best friend and top three friends* was assessed using the FQS.	*Depressive symptoms* were assessed using the Short Moods and Feelings Questionnaire [154].	Friendship quality of best friend was negatively associated with later depressive symptoms (β = − 0.72, 95% CI: − 0.97 - -0.47, *P* < 0.001). Friendship quality of top three friends friend was negatively associated with later depressive symptoms (β = − 0.69, 95% CI: − 0.94 - -0.44, *P* < 0.001).	14
Zhao et al., [95]	China	Cross-sectional	Setting: School *N* = 1863 Participants were categorized into three groups bases on their parents’ migration status: (i) Nonparent migrant group: *N* = 643 (49.6% female), age (mean) = 14.34 (SD) = 0.87 (ii) Both-parent migrant group: *N* = 409 (45.7% female), age (mean) = 14.33 (SD) = 1.19 (iii) Father-only migrant group: *N* = 750 (46.8% female), age (mean) = 14.32 (SD) = 0.84	*Friendship quality* was assessed by using the Chinese version of FQQ.	*Depressive symptoms* were assessed by using the Chinese version of the CDI	Friendship quality was negatively correlated with depressive symptoms among all adolescents’ groups irrespective of their fathers’ migration status (Nonparent migrant: r = − 35, both-parent migrant: r = −,32, Father-only migrant: r = −.29, *p* < .001)	10
Luijten et al., [96]	Netherlands	Longitudinal	Setting: School *N* = 1298 (53.2% female) Age (mean): 13.7 (SD: 1.1)	*Friendship quality* was assessed using the Network of Relationships Inventory.	*Wellbeing* was assessed using MHSC-SF.	No significant longitudinal association was found between friendship quality (T1) and adolescents’ wellbeing (T2)	12
Gautam et al., [97]	Nepal	Cross-sectional	Setting: School *N* = 371 (49.1% female) Age (mean): 17.4 (SD = 0.92)	*Relationship with friend* was assessed using The Patient Health Questionnaire (PHQ-9 [155];	*Depressive symptoms* were assessed using the PHQ-9.	Poor relationship with friend was significantly associated with higher depressive symptoms (β = 2.371, 95% CI: 1.078–5.215, *p* = 0.032)	11
Schwartz-Mette et al., [98]	United states	Longitudinal	Setting: School and community *N* = 186 (69.9% female) Age (mean): 15.68 (SD = 1.49)	*Same-gender positive friendship quality* was assessed using the revised revision of FQQ [156].	*Depressive symptoms* were assessed using CES-D.	Longitudinal bidirectional relationship between same-gender positive friendship quality and depressive symptoms was observed Significant inverse relationships were found between positive friendship quality (T1) and depressive symptoms (T2) (β = − 0.15, *p* < 0.000), and the inverse, to a lesser extent, was also true (β = − 0.08, *p* = *p* < 0.05)	12

+ve: Study found significant positive association

-ve: Study found significant negative association

Null: Study found no significant association

### Friendship quality and depressive symptoms

Of the twenty-three studies that looked at the impact of the quality of the relationship between peers on developing of depressive symptoms among adolescents, sixteen studies investigated that relationship cross sectionally [4, 57–67, 88, 93, 95, 97] and six studies utilized longitudinal design [83–86, 92, 94, 98], while case-control study has been employed in one study [56]. The quality of the included studies is of great concern as the methodological quality index of the included studies varies widely and ranges from six to fourteen. Besides, nine studies had weak analysis plan that does not extend to regression analysis [4, 57, 61, 65–67, 88, 93, 95]. Gender ratio imbalance was also noticed in one study [62]. For these reasons, the results must be interpreted with caution.

Assessment of the studies indicated that there is an observed consistency in the findings across all but two cross-sectional studies which suggests a beneficial association between better peer relationship and depressive symptomology during adolescence, but this consistency was not observed in the results of the six longitudinal studies as they varied in their conclusions. Of these six studies, two methodologically-sound studies supported the evidence of presence of a beneficial association between better peer bonds and lower depressive symptoms [83, 86, 94]. On the other hand, one short-term study demonstrates null effect of peer relationships on depressive symptoms [85]. One study suggested a direct impact only for poor peer relationships and higher depressive symptoms [84], while two studies revealed a bidirectional relationship between friendship quality and depressive symptoms [92, 98]. Therefore, based on the limitations and variations of the studies’ findings, high certainty of whether enhancing of friendship quality is of great benefit to prevent depression among teens could not be achieved.

### Friendship quality and loneliness

The association between friendship quality and perception of loneliness during adolescence was cross-sectionally examined in eight studies [62, 67–72, 93] and two case-control studies [55, 56]. The methodological quality index ranges from seven to fifteen. Irrespective of the quality of evidence, the replicability of the same results and conclusions across different populations, stages of adolescence, types of friendship quality in respect to gender and whether it is between best friends only, all supported the hypothesis that positive peer relationships were associated with lower levels of loneliness [55, 62, 67–71, 93]. However, this claim might not apply to adolescents with Asperger’s syndrome and those who live in rural areas as was highlighted in two studies [56, 72]. Furthermore, whether loneliness is more likely to affect those who experience non-ideal relationships with their peers or loneliness negatively impact friendship quality could not be judged as there is a lack of longitudinal studies in this area.

### Friendship quality and life satisfaction

Seven cross-sectional [73–77, 89, 91] and one longitudinal studies [90] have been identified and included into our review that assessed whether good peer relationship is associated with adolescents’ perception of life satisfaction. The quality index score of these studies ranges from eight to thirteen. Although the methodological quality index of the included studies does not vary significantly, major weaknesses were noticed in two studies which had an imbalance in gender ratio [75, 76], while one study drew its conclusion from the results of the univariate analysis [76]. However, the findings were homogenous across all the studies and suggested a significant association between experiencing a healthy relationship and adolescents’ life satisfaction.

### Friendship quality and happiness

Five included studies assessed whether happiness level is associated with maintaining a positive relationship with peers. This association was cross-sectionally examined in four studies [67, 70, 76, 81] while a longitudinal design was utilized in one study [83, 86]. The methodological quality index ranges from eight to thirteen. Two studies were poorly designed as both utilized weak statistical analysis method [67, 76] and one of them had gender ratio imbalance [76]. However, all of the included studies support the claim that better peer relationship is associated with higher levels of happiness among adolescents. Besides, the directionality of the relationship between friendship quality and happiness can be assumed, with low confidence, as this aspect was tested longitudinally in one study.

### Friendship quality and self-esteem

Six studies utilized cross-sectional analysis were carried out to investigate the association between quality of relationship and self-esteem during adolescence [66, 67, 78–80, 87, 93]. In regard to the methodological quality index, no major variation in the quality scores were noticed as the scores range from seven to ten. Five studies assumed that good companionship is associated with better self-esteem perception among teens [66, 67, 78, 79, 93], while only one study suggests the opposite [80, 87]. Although the results of majority of the studies are consistent, the directionality of the relationship cannot be confirmed due to lack of longitudinal evidence.

### Friendship quality and subjective wellbeing

There is a little evidence regarding the influence of quality of relationship in relation to subjective wellbeing during adolescence. One longitudinal [96] and four cross sectional studies [78, 79, 82, 91] were included with the methodological quality index ranging from nine to twelve. One of these studies was specific for adolescents aged 13 [82], while one small-scale study had imbalance in gender ratio [78]. The findings are consistent across the studies and suggested that healthy peer relationship is associated with a better perception of subjective wellbeing. However, due to the scarcity of studies in this area, especially longitudinal studies, generalization of this finding is not encouraged, nor directionality of the relationship can be established.

## Discussion

This systematic review included 43 studies investigating the association between friendship quality and six subjective wellbeing outcomes in adolescents [4, 55–98]. More than half of these studies [4, 56–67, 83–86, 88, 92–95, 97, 98] focused on depressive symptoms as their main wellbeing outcome, which reflect the shortage of studies in other domains of wellbeing outcomes. This shortage was more evident in the area of quality of peer tie in relation to happiness and subjective wellbeing, where only five studies for each have been found [67, 70, 76, 78, 79, 81–83, 86, 91, 96]. Beside this shortage, the cross-sectional design that have been used for most of the studies precludes us from reaching to more conclusive answers. Therefore, the interpretation of these results should be approached with caution. However, different conclusion and limitations for each relationship investigated can be drawn.

The evidence is indicative of an association between peer relationship quality and depressed mood [4, 56–67, 83–86, 88, 92–95, 97, 98]. Although there are concerns regarding the quality of the cross-sectional studies that assessed this relationship, the replicability of the same conclusion across studies increased the reliability of the evidence, which suggest that poor mood is related to poor friendship quality. Consistent with the finding, a review conducted by Roach [157] have found that support from friends has a beneficial buffering effect on poor mental health outcomes including depressive symptoms in adolescents, especially for those who are not in optimal mental health status. However, their review did not provide a causal explanation of how this mechanism occurs and it was only related to the influence of social support, not the potential effect of friendship quality. Moreover, the directionality of this relationship remains unclear due to the limited number of longitudinal studies, and the lack of consistency in the findings of the longitudinal studies. The ambiguity regarding how this mechanism occurs has several explanations. The stress prevention model developed by Gore [158] suggests that individual’s exposure to negative stressors can be decreased or prevented by the presence of support from close ones. Another model, called stress-buffering model, suggests that social support operates as a moderator between stress and negative mental health outcome, in which the individual’s ability to cope with difficulties is enhanced for those who have a better social support, as their interpretation of stressful life events they may experience are influenced positively in a pathway called cognitive appraisal process [159, 160]. The conflicting findings of the longitudinal studies in this review neither confirm nor deny this claim. Therefore, this arena of research needs further investigation. More longitudinal studies are needed to address the temporality concern. Studies on mediators and moderators are also required to address the ambiguity around the relationship between peer relationship quality and depressive symptoms. Hence, the reliance on this evidence alone while designing an intervention to reduce the prevalence of depression may not serve the purpose. A novel approach that avoids the shortcomings in the previous studies is required to fill this gap in the evidence base and uncover the mechanism that underlies the potential association between friendship quality and depression to help develop suitable interventions.

The studies included in this review regarding the association between friendship quality and loneliness [55, 56, 62, 67–72, 93] indicated that adolescents who experienced a higher friendship quality score reported less loneliness than their peers with lower quality friendships. A previous review identified the presence of intimacy in friendship as one of the important factors on lowering loneliness level in older adults [161]. The Belonging Hypothesis developed by Baumeister and Leary [162] suggests that searching for, and maintenance of, secure social relationships is part of our psychological formation as a social being (p. 497). Indeed, friendship formation itself satisfies our needs for social interaction with others and limits our sense of loneliness. Having a strong friendship would also limits this sense further. Interventions that focus on improving social skills and social support are one of the successful strategies that have been used to alleviate loneliness among adult population, as discussed in a previous meta-analysis [163]. This indicates that an individual with strong social skills can develop healthy friendships and hence few less lonelily than others. However, our conclusion from the included studies would have been of great value if it was supported by high-quality longitudinal evidence as there is a concern regarding the temporality of the associations observed. Therefore, this evidence should be interpreted with caution as it is only useful in developing a hypothesis that needs to be further tested.

This review also found a positive association between positive adolescents’ bonds and life satisfaction. A review conducted by Proctor et al. [164] discussed the value of social support from parents and friends to life satisfaction among youths. Their review showed that middle and late adolescence is the stage in which adolescents begin to rely more on their friends for social support. As human beings, non-material social assistance from friends and significant others is a need that when fulfilled can significantly impact on our perception of life satisfaction as shown in several researches [165–168]. However, considering that all, but one [90], of the studies included in this review are cross-sectional in nature [73–77, 89, 91], temporality could not be implied with high certainty. Further studies with a longitudinal design are needed to address the directionality concern, increase the validity of evidence, and better understand the mechanism that governs this relationship.

Our review supports the hypothesis that establishing a good relationship with peers can contribute significantly to better perception of happiness among adolescents. This beneficial association was observed cross-sectionally [67, 70, 76, 81] and longitudinally [83, 86]. This suggests that happiness level might be improved by investing on developing healthy friendship among adolescents. Along with our finding, a previous review conducted by Garcia et al. [169] reached the same conclusion. However, their work was limited to Latin American population and included all age groups. Therefore, considering the limited number of studies in this area and that almost half of these studies were conducted in New Zealand, one should not overemphasize or generalize this finding to all population.

This review also supports the hypothesis that healthy friendships can play a role in the adolescents’ perception of self-esteem and wellbeing. Almost all studies that have been found observed a positive association [66, 67, 78, 79, 82, 91, 93]. Previous review conducted by Gorrese and Ruggieri [170] on adolescents and young adults concluded that self-esteem can be boosted by secure peer relationships, especially if by friendships characterized by high level of trust and positive communication. This suggests that individuals’ sense of worthiness is partly formed and developed by perception of the quality of their social relationships, with better outcome for those with better friendship quality. However, there is a scarcity of studies, particularly longitudinal evidence, in this area. Further studies of good quality should be undertaken to better assess this relationship as the level of evidence obtained from those studies is not strong enough to reach a conclusive answer.

### Strengths and limitations

The association between friendship quality and subjective wellbeing in adolescents has been addressed for the first time in our review. A range of important subjective wellbeing constructs relevant to adolescents have been considered in this review to offer a broader understanding of this area of research. However, this work is not without limitations.

The first limitation is that no language other than English has been considered in the inclusion criteria due to time restriction. Hence, the generalizability of our findings could be limited for non-English-speaking countries because some of the evidence generated in these places might have published in other languages. Second is the absence of a meta-analysis component in our synthesis of evidence. That is because of poor reporting of the results in majority of the included studies as only twelve studies, for different wellbeing outcomes, have reported the effect estimate with the standard error, while other studies did not offer it nor provide any measures from which we could obtain it, such as the exact *p*-value or confidence intervals. The synthesis of our findings would have been improved by the reporting of these measures.

### Implications for future research and practice

There are two major gaps in the literature that future research should address: scarcity of longitudinal studies, and absence of studies on moderators and mediators – except in one study of mediators [58] – that underlies the association between friendship quality and subjective wellbeing. Therefore, future research should be more longitudinal in nature [21]. Moreover, there is a need for using advanced statistical analysis method [21], such as structural equation modeling and social network analysis, in order to better understand how quality of relation between friends impacts subjective wellbeing. Uncovering the underlying mechanism of this association and identification of the intermediary variables can be achieved by using structural equation modeling or similar approaches in which indirect mediational paths can be discovered. The difference in the association between study subjects in general and also based on their locations, such as their schools or communities, can also be accounted by using multilevel structural equation modeling. Social network analysis, on the other hand, can help visualizing the full friendship network structure and understanding how different patterns or levels of friendship between individuals play a role in shaping their wellbeing outcomes. Specific surveys should be developed to capture network data, hence, the complete network structure with relationships between individuals can be drawn and then examined. Future research examining the relationship between friendship feature (e.g., closeness, intimacy, and trust) and subjective well-being can also benefit from employing these methods. This would contribute to a better understanding of how different friendship characteristics influence subjective well-being during adolescence.

In practice, adolescents should be educated regarding the interrelation between friendship quality and subjective wellbeing outcomes. The role that good-quality social life and other potential risk factors could play in affecting their wellbeing should be highlighted. Such an educational intervention should involve schools where an atmosphere of learning, discussion, and development of healthy friendship during adolescence can be provided. Interventions can also take place where youth also tend to come together, such as community centers and places of worships. Caregivers, teachers, and other role models should also get involved and encourage adolescents to seek and nourish good quality friendships. They can engage formally – e.g., by participating in delivering interventions, workshops, campaigns, and other activities – and informally to educate about, and incentivize, the development of healthy friendships.

## Conclusion

The results suggest potential positive association between healthy friendships and better perception of wellbeing outcomes. However, there is a considerable lack of longitudinal studies and studies of mediators and moderators that underlies this association. Further studies that employ study designs and analytical methods that are more suitable to investigate the causal relationship between friendship quality and subjective wellbeing constructs are needed.

## Supplementary Information

**Additional file 1.** PRISMA checklist.

**Additional file 2.** PRISMA abstract checklist.

**Additional file 3.** SWiM checklist.

**Additional file 4.** Methodological quality assessment.

## Data Availability

The datasets supporting the conclusions of this article are included within the article and its additional files.
